# Faecal microbiota transplantation for *Clostridioides difficile* infection: Four years’ experience of the Netherlands Donor Feces Bank

**DOI:** 10.1177/2050640620957765

**Published:** 2020-09-29

**Authors:** Elisabeth M Terveer, Karuna EW Vendrik, Rogier E Ooijevaar, Emilie van Lingen, Eline Boeije-Koppenol, Els van Nood, Abraham Goorhuis, Martijn P Bauer, Yvette H van Beurden, Marcel GW Dijkgraaf, Chris JJ Mulder, Christina MJE Vandenbroucke-Grauls, Jos FML Seegers, Joffrey van Prehn, Hein W Verspaget, Ed J Kuijper, Josbert J Keller

**Affiliations:** 1 Department of Medical Microbiology, Leiden University Medical Center, Leiden, the Netherlands; 2 Department of Gastroenterology and Hepatology, Amsterdam University Medical Centers, Amsterdam, the Netherlands; 3 Department of Gastroenterology, Leiden University Medical Center, Leiden, the Netherlands; 4 Department of Internal Medicine, Erasmus Medical Center, Rotterdam, the Netherlands; 5 Department of Internal Medicine, Amsterdam University Medical Centers, Amsterdam, the Netherlands; 6 Department of Internal Medicine, Leiden University Medical Center, Leiden, the Netherlands; 7 Department of Gastroenterology and Hepatology, Spaarne Gasthuis, Hoofddorp, the Netherlands; 8 Department of Epidemiology and Data Science, Amsterdam University Medical Centers, Amsterdam, the Netherlands; 9 Department of Medical Microbiology and Infection Control, Amsterdam University Medical Centers, Amsterdam, the Netherlands; 10 Unaffiliated; 11 Department of Biobanking, Leiden University Medical Center, Leiden, the Netherlands; 12 Department of Gastroenterology, Haaglanden Medisch Centrum, The Hague, the Netherlands

**Keywords:** Faecal microbiota transplantation, *Clostridioides difficile*, stool bank, donor, cure rate, microbiome, microbiota modifying therapy

## Abstract

**Background:**

The Netherlands Donor Feces Bank provides standardized ready-to-use donor faecal suspensions for faecal microbiota transplantation treatment of patients with recurrent *Clostridioides difficile* infection.

**Objective:**

The purpose of this study was evaluation of safety, feasibility and outcome of faecal microbiota transplantation facilitated by a national stool bank.

**Methods:**

The methods used included: observational cohort study of donors and recipients of faecal suspensions; assessment of donor screening and patient selection performed by an expert panel of medical microbiologists, gastroenterologists and infectious disease specialists; and patient outcome evaluated at different timepoints after faecal microbiota transplantation.

**Results:**

Of 871 volunteers who registered as a potential faeces donor, 16 (2%) became active donors. Nine donors stopped or were excluded after a mean donation period of 5.7 months. In 2016–2019, 47 (27%) of 176 requests for faecal microbiota transplantations were deemed not indicated by the expert panel. In total, 129 patients with recurrent *C. difficile* infection were treated with 143 faecal suspensions in 40 different hospitals. The cure rate at two months after a single infusion was 89% (107/120). Of 84 patients, long-term follow-up (median 42 weeks) was available and sustained cure was achieved in 61 (73%). Early *C. difficile* infection relapses (within two months after faecal microbiota transplantation) and late recurrences (after more than two months) occurred more frequently in patients who received non-*C. difficile* antibiotics within three weeks after faecal microbiota transplantation and in moderately to severely immunocompromised patients. Of 21 patients with *C. difficile* infection after faecal microbiota transplantation, 14 were cured with anti-*C. difficile* antibiotics and seven with a second transplantation. No faecal microbiota transplantation-related serious adverse events were observed, but gastro-intestinal complaints (nausea, abdominal pain or diarrhoea) persisted in 32% of the treated patients at long-term follow-up.

**Conclusion:**

Faecal suspensions provided by a centralized stool bank, supported by a multidisciplinary expert team, resulted in effective, appropriate and safe application of faecal microbiota transplantation for recurrent *C. difficile* infection.

**Level of evidence:**

Level II, prospective cohort study

## Key Summary

### Established knowledge on this subject

- Faecal microbiota transplantation (FMT) is an established therapy for multiple recurrent *Clostridioides difficile* infection (rCDI).
- Only a small percentage of potential donors are eligible after careful selection and screening.
- Centralized stool banks provide an opportunity for quality improvement of FMT.

### Significant and/or new findings of this study?

- FMT that is facilitated by a national stool bank, is efficacious, safe and appropriately used.
- Consultation by a multidisciplinary FMT-expert team results in appropriate use of FMT.
- Post-FMT *Clostridioides difficile* infection (CDI) relapse can be treated with antibiotics directed against CDI, even if these were ineffective prior to FMT in those patients.
- Faecal suspensions for rCDI treatment can be stored at –80°C for up to two years, without loss of effectiveness.

## Introduction

Faecal microbiota transplantation (FMT) is a very effective treatment for recurrent *Clostridioides difficile* infection (rCDI). In recent years it has been implemented worldwide as an effective rescue therapy with cure rates of approximately 85%.^1–4^ Transplanting faecal microbiota of a healthy donor with the aim of restoring a patient’s perturbed microbiota also appears promising for several other disorders, such as ulcerative colitis and hepatic encephalopathy.^5^,^6^ Careful donor screening is required, minimizing the risk of pathogen transfer or an impaired microbiota composition potentially predisposing for disease. With the emergence of FMT as a new treatment approach, stool banks are needed to provide ready-to-use donor faecal suspensions that are produced in a standardized way.^7^ Significant advantages of centralized donor screening and production of donor faecal suspensions are the possibilities of providing quality assurance, and appropriate monitoring of potential yet unknown adverse events.^7^

At present, stool banks operating at an institutional level exists in several countries, and national operating stool banks are active in the USA, the Netherlands and England.^8–12^ In 2015, the Netherlands Donor Feces Bank (NDFB) was founded as a non-profit national stool bank. In addition to providing faecal suspensions, the NDFB provides advice on the diagnosis, treatment and follow-up of recurrent or severe *Clostridioides difficile* infection (CDI) by an FMT-expert panel of medical microbiologists, gastroenterologists and infectious disease specialists. The NDFB expert panel evaluates each request for FMT.^10^

The aim of the current evaluation report was to describe the results of donor screening and the outcome of FMT performed for rCDI facilitated by the NDFB, and under guidance of its expert panel. In addition, donor-, patient- and faecal suspension-specific factors underlying FMT treatment failure are addressed.

## Material and methods

### Study design

This was a prospective, observational cohort study describing the results of faeces donor screening and patient outcome after FMT from the first performed donor screening in January 2016, and FMT in May 2016, until August 2019.

### Screening and selection of donors

The NDFB recruits healthy, unrelated volunteers who can supply stool to the to the microbiology laboratory within two hours after defaecation. The procedure of donor recruitment, screening by questionnaire, interview and laboratory testing was described before,^10^ and is summarized in Supplementary Material Table S1.

### Processing and storage of faecal suspensions

The NDFB uses standardized procedures for collection, preparation and storage of donor faecal suspensions.^10^ In short, 60 g of donor faeces is used for the preparation of one faecal suspension. Storage at –80°C is accommodated by a certified biobanking facility. At the NDFB, the maximum shelf-life has been (arbitrarily) determined as two years.

### FMT consultation and treatment

Requests for faecal suspensions are submitted to the NDFB by treating physicians using a standardized form (www.ndfb.nl). The request is evaluated by at least three medical specialists (a gastroenterologist, medical microbiologist and infectious disease specialist) of the NDFB expert panel. The indication for FMT is assessed, the diagnosis of rCDI is verified, and the feasibility and safety of FMT for the individual patient is considered. Patients with at least two recurrent CDI episodes or severe and therapy refractory CDI are eligible. CDI is defined as diarrhoea (≥3 unformed stools per 24 h for two consecutive days; or ≥8 unformed stools per 48 h), in combination with a positive diagnostic test for *C. difficile* and absence of another more likely cause of diarrhoea. To differentiate between infection and asymptomatic colonization, a two-stage testing algorithm is recommended.^13^ In particular, presence of free *C. difficile* toxins is a prerequisite for patients with gastro-intestinal comorbidity. Severe CDI is defined by the presence of severe colitis or a complicated course, with systemic toxin effects and shock that may result in ICU admission or colectomy.^14^

If a patient is eligible for FMT, a donor faecal suspension is transported to the requesting hospital on dry ice and thawed according precise instructions.^10^ In general, prior to FMT, patients receive vancomycin (125–250 mg four times a day) for a minimum of 4 days until 24 h pre-FMT. For duodenal delivery, 2l of KleanPrep (bowel lavage) is prescribed 1 day prior to FMT.^10^ Treating physicians are instructed how to perform FMT. The thawed faecal suspension is infused through a duodenal tube, at an advised rate of 10 cc/min. If FMT through a duodenal tube is considered unsafe or contra-indicated (i.e. due to a hampered bowel passage or increased aspiration risk), infusion via colonoscopy is advised. After infusion of the donor faeces, patients are monitored for 2 h.^1^,^10^ Antibiotic stewardship to protect the microbiota post-FMT is advocated to prevent a relapse of CDI after FMT.^10^,^15^,^16^

### Follow-up

At each FMT treatment, the patient and treating physician receive information on potential adverse events and are advised to contact the NDFB if such an event occurs. Treating physicians are advised to plan a routine follow-up visit at 3 weeks post-FMT and patients are requested to complete a questionnaire. Patients are routinely approached by an NDFB employee by telephone 2 months after FMT, and for the present evaluation report also at a later time-point between January 2019–August 2019 (19–143 weeks) post-FMT for long-term follow-up. Information about recurrence, hospital admission, possible FMT-related adverse events and antibiotic use is collected. We defined early relapse as a CDI episode within two months following FMT,^14^ whereas a CDI episode after two months post-FMT was regarded as late recurrence. We defined cure as resolution of all CDI symptoms, and no CDI relapse within three weeks (primary cure), two months (cure at two months) or long-term follow-up (sustained cure). We categorized the relationship between adverse events and FMT as follows: definitely related, probably related, possibly related and unrelated to FMT.^17^

### Statistical analysis

The statistical analysis was performed using SPSS 23.0 statistical software. Continuous data are presented as mean (range), or median in the case of a skewed distribution. Possible associations between FMT treatment outcome and patient, faecal suspension or donor characteristics were tested by a Pearson’s Chi-squared test or Fisher’s exact test, where appropriate. An odds ratio was calculated using logistic regression and presented with a 95% confidence interval (95% CI). For ordinal data, a linear-by-linear association test was used. Kaplan-Meier curves and log-rank tests were performed to assess CDI-free survival. A two-tailed significance level of *p*<0.05 was considered statistically significant. Missing data and patients lost-to follow-up were mentioned but data was not corrected for this.

## Results

### Donor selection and screening

Since the initiation of the NDFB, 871 volunteers registered as potential faeces donors. After receiving information about donor requirements, 603 withdrew and 268 completed an online questionnaire (Table 1). Based on the questionnaire, 83 (31%) donors were invited for an interview, followed by microbiological testing. After evaluation of the interviews, screening and rescreening of faeces and serum, only 16 volunteers were eligible as faeces donors, which is 6% of volunteers completing the questionnaire and 2% of all initially interested individuals (Table 1). Of these 16 active donors, 10 (63%) were female, the mean age was 33 (range 24–57) years, and the mean body mass index (BMI) was 22.4 (range 19.6–24.8) kg/m^2^. Asymptomatic, transient carriage of potential pathogens was occasionally found at re-screening (multidrug-resistant organism (MDRO): *n* = 4, norovirus: *n* = 2, rotavirus: *n* = 1, sapovirus: *n* = 1, parechovirus: *n* = 1, *Salmonella* species: *n* = 1, or *Dientamoeba fragilis*: *n* = 1). Nearly all active donors experienced one or more transient episodes with upper respiratory complaints, diarrhoea, temporary change of defaecation pattern or antibiotic use, for which donations were temporarily stopped. Nine of the 16 (56%) donors stopped or were excluded after a mean period of 5.7 months (range 1–14 months). Reasons for discontinuation were persistent carriage of potential pathogens during repeated testing (*Blastocystis* species: *n* = 2, MDRO: *n* = 1, or *D. fragilis n* = 1) or a too heavy burden of required time and logistics (*n* = 5).

**Table 1.** Results of the donor selection and screening process.

Donors (%)	Action	Excluded (%)	Exclusion reasons^a^
871	Request for more information by donor	603 (69%)	52% (*n* = 311) withdrawal after reading additional information, 22% (*n* = 132) unable to deliver faeces 2 h after defaecation, 20% (*n* = 121) age >50 years,^b^ 8% (*n* = 49) increased risk disturbed microbiota (bowel complains, medication use, comorbidity, depression, BMI>25 m^2^/kg, etc.), 5% (*n* = 29) other
268 (100%) ↓	Donor fills-out an extended questionnaire	185 (69%)	22% (*n* = 41) comorbidity/medication use, 22% (*n* = 40) BMI<18.5 or >25 m^2^/kg, 18% (*n* = 33) (history of) depression, 15% (*n* = 28) profession of healthcare worker,^c^ 14% (*n* = 25) age >50 years,^b^ 14% (*n* = 25) bowel complaints, 12% (*n* = 22) inability to deliver faeces <2 h, 10% (*n* = 19) withdrawal after completing questionnaire, 6% (*n* = 12) (close relative with) IBD, 5% (*n* = 9) frequent travelling, 4% (*n* = 7) risk factor for colon carcinoma,^d^ 3% (*n* = 6) high risk sexual behaviour, 7% (*n* = 13) other
83 (31%) ↓	Interview	17 (20%)	65% (*n* = 11) donors withdrawal or failure to deliver faeces <2 h once a week, 35% (*n* = 6) donors excluded based on interview (IBS complaints, comorbidity, psychological evaluation, patient contact, atopy)
66 (25%) ↓	Faeces^e^ screening	47 (71%)	89% (*n* = 42) *Dientamoeba fragilis*, 15% (*n* = 7) MDRO, 9% (*n* = 4) *Blastocystis* sp., 4% (*n* = 2) *Helicobacter pylori*, 2% (*n* = 1) *Campylobacter jejuni*, 2% (*n* = 1) *Entamoeba histolytica*
22^f^ (8%) ↓	Serum screening	0 (0%)	None
22^f^ (8%) ↓ 16 (6%)	First rescreening and donor withdrawalActive donor	6 (27%)	Exclusion of quarantined donor suspensions: 83% (*n* = 5) difficulty to implement donation in daily practice, 17% (*n* = 1) MDRO and refusal to perform rescreening

BMI: body mass index; IBS: irritable bowel syndrome; MDRO: multidrug-resistant organism.

^a^Some volunteers had multiple exclusion criteria, exclusion is displayed as the percentage of total excluded donors as result of a particular screening step.

^b^From September 2018 changed to 55 years, or 60 years with negative colon carcinoma screening.

^c^Higher risk of temporary carriership of pathogens.

^d^Close relative with colon carcinoma with an onset below the age of 50 years.

^e^Screening algorithm used: first screening includes: *Dientamoeba fragilis*, microscopy for *Blastocystis* sp., MDRO and *Helicobacter pylori* screening, if negative, then additional tests are performed (Supplementary Material Table S1).

^f^Three donors were excluded at first screening, successfully decolonized of MDRO, *D. fragilis* or *E. histolytica*, and they subsequently continued the donor screening program.

### FMT consultation

Since May 2016, 176 FMT requests for treatment of rCDI or therapy refractory CDI patients were reviewed by the expert panel. Of these requests, 47 (27%) were deemed not indicated. The most frequent reason for rejection was *C. difficile* carriership in combination with diarrhoea due to inflammatory bowel disease (IBD) or another, unknown cause. Detailed results of the evaluation of FMT requests are listed in Table 2.

**Table 2.** Results of the evaluation of faecal microbiota transplantation (FMT) requests by multidisciplinary FMT expert panel.

FMT decision	Number of requests
FMT request rejected by NDFB expert panel	47/176 (27%)
Reasons of rejection of the 47 FMT requests:	
*C. difficile* carriership and diarrhoea due to other cause;	30 (64%)
• Diarrhoea with unknown cause	–18
• Diarrhoea due to IBD	–12
Anti-CDI antibiotics advised instead of FMT;	11 (23%)
• First, mild recurrence	–7
• New CDI infection (too long interval between CDI episodes)	–4
Long-term antibiotic use/elective operation	3 (6%)
Withdrawal of FMT request after observed antibiotic treatment effect, by treating physician or patient	3 (6%)
Request for FMT approved by NDFB expert panel	129/176 (73%)
FMT indication^a^	
• Multiple recurrent CDI	125 (97%)
• Severe, therapy refractory CDI	3 (2%)
• Refractory CDI	1 (1%)

CDI: *Clostridioides difficile* infection; IBD: inflammatory bowel disease.

^a^One hundred and forty-three FMTs performed in 129 patients. Ten patients received multiple FMTs; nine patients for treatment of a post-FMT CDI relapse (seven patients cured with a single repeat FMT, one patient cured with two repeat FMTs, one patient cured with antibiotics after a repeat FMT) and one patient received sequential FMT treatment for severe, therapy refractory CDI (in total six FMTs; three FMTs for a first episode and three FMTs for the relapse).

### FMT treatment

In total, 129 patients with CDI were treated with 143 FMTs in 40 different hospitals throughout the Netherlands. Suspensions obtained from 12 of the 16 approved donors were used. The mean age of the patients was 69.9 years (range 2–96), and 60% were female. Patients suffered from a mean of 4.2 (range 1–10) CDI episodes before FMT was considered. Most patients had rCDI (Table 2). Four patients received an FMT for a first episode of severe, therapy-refractory CDI, of whom one received multiple FMTs (six in total). The majority of FMTs (127/143, 89%) were infused through a duodenal tube. FMT via the lower gastro-intestinal route was performed by colonoscopy because of motility disorders (*n* = 4), an already planned colonoscopy to rule out IBD (*n* = 8); or sigmoidoscopy because of an ileus due to severe CDI (*n* = 4).

### Outcome of FMT treatment

**Figure 1.**
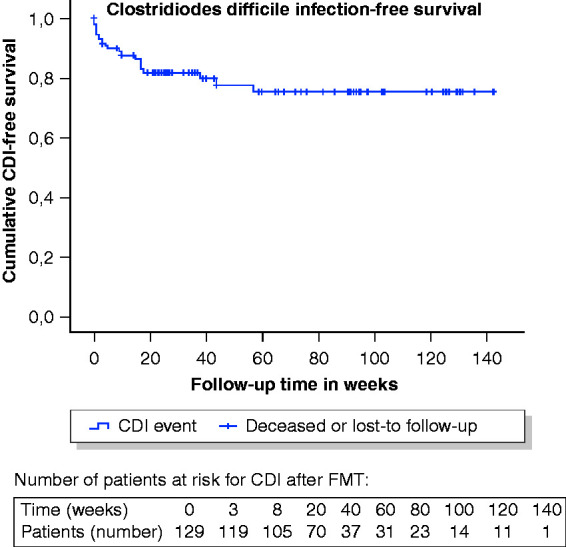
Kaplan-Meier curve of the *Clostridioides difficile* infection (CDI) (faecal microbiota transplantation (FMT) failure, early relapse or late recurrence)-free survival post-FMT.

Follow-up data were available for 128 of 129 patients at three weeks, and 120 patients at 2 months after FMT. Three patients (2%) died within 3 weeks due to causes unrelated to the FMT. The primary cure rate at 3 weeks after a single FMT infusion was 91% (117/128). Cure at 2 months post-FMT was 89% (107/120). Thirteen patients suffered from an early relapse at a median of 1 week (range 0–5 weeks) post-FMT. Of the 129 FMT-treated patients; 11 (9%) were deceased by the time of long-term follow-up, 34 (26%) were lost to follow-up. From 84 (65%) patients, long-term follow-up was available with a median of 42 weeks (range 19–143 weeks, interquartile range 26–97 weeks). Ten patients developed a late CDI recurrence after a median of 17 weeks (9–57 weeks) post-FMT. Thus, sustained cure was achieved in 61 of 84 (73%) patients still alive at long-term follow-up. Figure 1 shows the CDI-free survival over time. The 23 patients suffering from post-FMT CDI had symptoms of diarrhoea, either in combination with a positive toxin enzyme immuno assay (EIA) (14/23, 61%), polymerase chain reaction (PCR) (5/23, 22%), or were diagnosed with unclear methods but with clinical response to vancomycin treatment (4/23, 18%). Most patients experiencing CDI post-FMT eventually successfully cured (21/23), either by antibiotics alone (14/21, 67%; received fidaxomicin, four vancomycin, one metronidazole and one fidaxomicin) or by a second FMT (7/21, 33%; of whom one patient needed a third FMT). In two patients CDI treatment was not initiated, the patients died of an underlying disease with concurrent development of CDI.

### Risk factors for post-FMT CDI

Patients with an early CDI relapse post-FMT had more often received non-CDI antibiotics during the first 3 weeks after FMT compared to patients without relapse (39% versus 15%) (Table 3). Antibiotic use preceded a late CDI recurrence in 80% of patients. Nonetheless, antibiotic use shortly after FMT was still a significant predictor of all CDI episodes post-FMT (both early AND late) (40% (8/20) compared to 15% (14/95), *p*-value 0.001, Supplementary Material Figure S1). In addition, early CDI relapses were observed more frequently in patients who were moderately (3/23) to severely (2/3) immunocompromised (Table 3). A trend was observed towards more CDI (early relapse or late recurrence) post-FMT in immunocompromised (31%, 8/26) versus immunocompetent patients (15%, 15/101) (*p*-value 0.054, Supplementary Material Figure S2). No other patient or faecal suspension characteristic significantly differed between those who relapsed and those cured (Table 3). Importantly, a longer processing time of faecal suspensions (mean 168 min, range 65–355 min) or longer storage time at –80°C (mean 269 days, range 34–730 days, 30/129 were stored >1 year) did not negatively influence the success rate of FMT. Donor selection did not influence the outcome of FMT; no differences between donors could be detected (*p*-value 0.10, individual donor data in Supplementary Material Table S2).

**Table 3.** Patient, donor and faecal suspension risk factors for *Clostridioides difficile* infection (CDI) relapse within two months after faecal microbiota transplantation (FMT).

Characteristic	Patients with relapse within two months post-FMT	Patients cured at two months post-FMT	Results statistical analyses (OR (95% CI), p-value)
Patient sex (female)	77% (10/13)	58% (67/116)	OR 2.4 (0.6–9.3), *p*-value 0.24
Donor sex (female)	54% (7/13)	50% (58/116)	OR 1.2 (0.4–3.7), *p*-value 0.79
Donor – patient sex mismatch	39% (5/13)	47% (54/116)	OR 0.7 (0.2–2.3), *p*-value 0.58
Patient’s age (at FMT)	69 years (41–96)	70 years (2–92)	*p*-value 0.76
Donor’s age (at donation)	36 years (24–46)	35 years (24–46)	*p*-value 0.82
Lower gastro-intestinal infusion of FMT (sigmo- or colonoscopy)	23% (3/13)	8% (9/116)	OR 3.6 (0.8–15.3), *p*-value 0.10
Mean processing time of the faecal suspension (defaecation to freezer)	163 min	168 min	*p*-value 0.73
Mean storage time of the faecal suspension (at –80°C)	214 days	275 days	*p*-value 0.27
Severe CDI as indication for FMT	8% (1/13)	2% (2/116)	OR 4.8 (0.4–56.3), *p*-value 0.28
Prior CDI relapses, before FMT is performed	2.6 (13)	2.8 (114)	*p*-value 0.69
PPI use	61% (8/13)	51% (55/108)	OR 1.5 (0.5–5.0), *p*-value 0.47
Comorbidity of IBD	8% (1/13)	11% (13/114)	OR 0.7 (0.1–5.4), *p*-value 1.0
Severe kidney comorbidity: dialysis or kidney transplantation	8% (1/13)	9% (24/112)	OR 1.0 (0.1–8.2), *p*-value 1.0
rUTI in medical history	0% (0/13)	8% (9/113)	*p*-value 0.60^a^
Use of non-CDI antibiotics in between the prior CDI episodes	46% (6/13)	38% (43/113)	OR 1.4 (0.4–4.4), *p*-value 0.57
Immunocompromised^a^			
– Not	61% (8/13)	82% (93/114)	*p*-value 0.01
– Moderate	23% (3/13)	18% (20/114)	
– Severe	15% (2/13)	1% (1/114)	
Hypervirulent clade^b^	25% (2/8)	20% (13/65)	OR 1.3 (0.2–7.4), *p*-value 0.66
Post-FMT hospitalization for non-CDI indications post-FMT^c^	23% (3/13)	14% (14/102)	OR 1.9 (0.5–7.7), *p*-value 0.41
Post-FMT infection (other than CDI)^c^	15% (2/13)	17% (17/102)	OR 0.9 (0.2–4.5), *p*-value 1.00
Post-FMT antibiotic use (non-CDI indications)^c^	39% (5/13)	15% (15/102)	OR 3.6 (1.0–12.6), *p*-value 0.03

CI: confidence interval; IBD: inflammatory bowel disease; OR: odds ratio; PPI: proton pump inhibitor; rCDI: recurrent *Clostridioides difficile* infection; rUTI: recurrent urinary tract infection.

Percentages and final odds ratio with 95% CIs of FMT-treated patients with or without early CDI relapse.

^a^Immunocompromised classified as: not, moderate or severe. Patients are regarded as severely immunocompromised when: neutropenic, (scheduled or received last 100 days) an allogenic stem cell transplantation, active Graft-versus-host-disease requiring immunosuppressive agents, and moderately immunocompromised when: having <200 CD4 T-cells/µl, prolonged use of corticosteroids at a mean dose of 0.3 mg/kg/d of prednisone equivalent for >3 weeks, treatment with other recognized T-cell immunosuppressants during the last 90 days or have an inherited severe immunodeficiency.

^b^Hypervirulent clade RT027 (016, 019, 0247, 036, 075, 111, 112, 153, 156, 176, 208, 273) and clade RT078 (033, 045, 066, 078, 126, 127).

^c^In the first 3 weeks post-FMT.

### Patients: follow-up of adverse events

On the day of FMT, 66% (62/94) of patients had mild, transient gastro-intestinal complaints (Table 4). At 3 weeks and at long-term follow-up, a subset of patients still reported abdominal pain (both 21%) and diarrhoea (27% and 33%, respectively). The self-rated defaecation pattern after FMT compared to the pre-existent defaecation pattern (before the CDI episodes) had improved in 16% at 3 weeks, and in 38% at long-term follow-up (Table 4).

**Table 4.** Gastro-intestinal complaints post-faecal microbiota transplantation (FMT).

Gastro-intestinal complaint	Day of FMT^a^	1-week post-FMT^a^	3-weeks post-FMT^a^	LTFU^b^
Nausea (% yes)	20% (19/94)	14% (13/96)	11% (11/97)	18% (13/73)
Abdominal pain (% yes)	33% (31/93)	28% (27/97)	21% (21/98)	21% (15/71)
Diarrhoea (% yes)	52% (48/93)	30% (29/97)	27% (26/97)	33% (24/73)
Self-rated defaecation pattern (post-FMT vs before CDI episode)	n/a	n/a		
• Improved			16% (13/80)	38% (25/65)
• Similar			68% (54/80)	46% (30/65)
• Deteriorated			16% (13/80)	15% (10/65)

LTFU: long-term follow-up; rCDI: recurrent *Clostridioides difficile* infection.

^a^A questionnaire is filled in by the patient or treating physician at regular follow-up 3–4 weeks post-FMT.

^b^LTFU: median 42 weeks, range 19–143 weeks.

No definitely or probably related serious adverse events were reported. Five (5/128, 4%) FMT (procedure)-related adverse events were observed (Table 5). Regurgitation of donor faeces occurred in four patients shortly after duodenal infusion of the faecal suspension (Table 5). During the first three weeks after FMT, 23% (26/115) of the patients were admitted to the hospital or had prolonged hospitalization, of which nine (8%) for possibly FMT-related indications (Table 5). The most frequently observed infections after FMT were urinary tract infection (UTI) (8%, 9/115) or pneumonia (5%, 6/115). The majority of patients suffering of these infections had known predisposing factors for UTI or pneumonia (Table 5).

**Table 5.** (Serious) adverse events (AEs) within three weeks after faecal microbiota transplantation (FMT).

Description adverse event		Number of patients
Definitively or probably related to FMT		
SAE	None	0% (0/128)
AE	Procedure-related AEs– Regurgitation, no aspiration, patient successfully treated– Sore throat after placing duodenal tube	4% (5/128) – 4– 1
Possibly related to FMT		
SAE	Hospitalization within 3 weeks post-FMT due to: – Lower respiratory tract infection (causing pathogen unknown)^a^– Urinary tract infection (causing pathogen unknown)^b^– Diarrhoea (non-CDI)	8% (9/115) – 5– 3– 1
AE	Gastro-intestinal (see Table 4) Infections– Urinary tract infection (causing pathogen unknown)^b^– Urinary and lower respiratory tract infection^a^Other– Fever– Possible flare IBD (ulcerative colitis), uncertain if it was pre-existent	11–52%– 5– 1– 1– 1
Unrelated to FMT		
SAE	Hospitalization (or prolonged hospitalization) within 3 weeks post-FMT– Lower respiratory tract infection (COPD exacerbation due to *Moraxella catarrhalis* and RSV infection) – CDI relapse– Related to pre-existent comorbidity (elective surgery dialysis shunt, complications knee prosthesis, perforated diverticulitis, hyponatraemia with tongue carcinoma, GvHD after allogenic stem cell transplantation (already existing), diarrhoea due to chemotherapy) – Death within 3 weeks post-FMT due to comorbidity (tongue carcinoma/hyponatraemia, sepsis due to pneumonia, comorbidity GvHD lung after allo-SCT) – Infection with *Yersinia pseudotuberculosis* post-FMT, donor suspension tested negative (with both PCR and culture (cold enrichment-broth) – Perforated diverticulitis, in retrospect already present before FMT (upper GI delivery) – CVA	15% (17/115) – 2– 3– 6– 3– 1– 1– 1
AE	Infections– Otitis, infection of toe, phlegmon groin	- 3

CDI: *Clostridioides difficile* infection; SAE: serious adverse event; UTI: urinary tract infection; COPD: chronic obstructive pulmonary disease; CVA: cerebro vascular accident; GI: gastrointestinal; GvHD: graft versus host disease; PCR: polymerase chain reaction; RSV: respiratory syncytial virus; SCT: stem cell transplantation.

^a^Four patients (80%, 4/5) developing a pneumonia had a medical history of either chronic obstructive pulmonary disease, asthma or lung fibrosis.

^b^Four patients (44%, 4/9) had known predisposing factors for UTI (medical history of pyelonephritis, diabetes type II and benign prostate hypertrophy, Sachse urethrotomy or Bricker bladder).

## Discussion

During the 4 years since its establishment, the NDFB has evaluated over 175 FMT requests and provided standardized FMT to almost 130 patients affected by rCDI. A high cure rate of nearly 90% at 2 months after FMT and a sustained cure rate over 70% at 42 weeks post-FMT was observed.

The cure rate of FMT facilitated by the NDFB appears high compared to the 76% cure rate reported in a recent meta-analysis of single FMTs for rCDI.^4^ This may be explained by the stringent criteria for diagnosis and treatment applied by our FMT expert panel.^13^ This expert panel discusses the indication for FMT and provides advice during treatment and follow-up of the patients. We rejected a quarter of FMT requests, mainly because the diarrhoea was attributed to another cause that coincided with *C. difficile* carriership. Thus, consultation might prevent inappropriate use of FMT and increases the clinical benefits and cost-effectiveness. Our observation is similar to a previous report from an FMT-expert centre, which showed that 25% of patients referred for FMT did not have confirmed rCDI.^18^ In particular, new onset or persistent activity of IBD appears to be a diagnostic pitfall.^18^,^19^ In addition, non-responsiveness to anti-CDI antibiotics seems to point to an alternative diagnosis rather than therapy-refractory CDI in most patients. In fact, only four of our 129 patients were deemed to suffer from therapy-refractory CDI by the expert-panel.

A subgroup of rCDI patients remains vulnerable for CDI after FMT, as 9% (10/107) of initially cured patients developed a late CDI recurrence. Of this group, 80% had used antibiotics preceding the recurrence, in contrast to only 39% of patients with an early relapse. This indicates that antibiotic use after FMT should be limited as much as possible for a prolonged period. It also emphasizes that a long follow-up after FMT is mandatory to assess the long-term efficacy of FMT. The majority (61%) of early relapses were not preceded by antibiotics, indicating that other factors also contribute to FMT failures, such as an immunocompetence. Other studies have identified Charlson Comorbidity Index,^20^ the severity of CDI,^21^,^22^ previous (CDI) hospitalisation,^22^,^23^ inpatient status,^22^ surgery,^23^ female sex^23^ and older age^24^ to predict recurrence after FMT. We did not recognize donor-related factors contributing to FMT outcome, confirming previous reports.^25–27^ The majority of patients with post-FMT CDI were cured with antibiotic treatment, suggesting that this should be considered a different entity compared to the antibiotic resistant episodes prior to FMT. This could be explained as an FMT-mediated gut microbiota reset, which renders patient less susceptible to rCDI after treatment with antibiotics alone.

In our experience, duodenal delivery of donor faeces was highly effective and certainly not inferior to delivery by colonoscopy, although this report was not designed as a study to compare routes of delivery. Duodenal FMT has a small risk of regurgitation. To prevent this, we currently advise slow infusion of the faecal suspension in the duodenum (10 cc/min), room temperature of the suspension to avoid cold shock, and colonoscopic delivery in case of possible bowel dysmotility. After introduction of these precautions, regurgitation was no longer recognized. We did not observe FMT-related serious adverse events. Several patients developed a UTI (9/115) or pneumonia (6/115) after FMT. This might be explained by existing predisposing factors in most patients, although a relationship with FMT cannot be fully excluded. Interestingly, it has been suggested that the incidence of UTI could decline after FMT due to a reduced abundance of *Enterobacterales* in the gut.^28^ About 21–33% of patients suffered from abdominal complaints at follow-up. This is in line with a previous report, in which no FMT-attributable factors could be identified.^29^ Remarkably, at long-term follow-up, the self-rated defaecation pattern improved (38%), or had stayed unchanged (46%) in most of our patients, compared to the period before the first CDI episode, suggesting that gastro-intestinal symptoms after FMT could be related to post-infectious complaints and pre-existent comorbidity. Post-infectious irritable bowel complaints after CDI were also reported in 4–25% of patients not given FMT.^30^

We observed a low 2 months mortality rate of 3% (3/120) after FMT, which is lower than the 30-day mortality rate of primary CDI in the Netherlands^31^ (9% overall mortality). The mortality of 12% at long-term follow-up (median 42 weeks) is lower than observed in two other FMT cohorts (20% at weeks 30 and 48 post-FMT).^32^,^33^

A strength of our evaluation report is the structural follow-up of donors and patients of a complete stool bank cohort, with use of standardised questionnaires and over a long period of time post-FMT. Several studies report on retrospective analyses of only specific patient groups treated with stool bank FMT-suspensions without structural long-term follow-up. In one of the largest retrospective studies, 307 of 528 (39%) rCDI patients were successfully contacted, a sustained cure of 76% at 34 months follow-up was observed.^33^ Our high sustained cure rate confirms this observation. A limitation of our report is that 26% patients were lost to long-term follow-up, and late recurrent CDI may be overestimated as these were actively reported to the NDFB by the local physicians. This is supported by the fact that no unreported recurrences were detected with the follow-up questionnaires. Another limitation, also related to the setting of a national stool bank, is the lack of nation-wide uniform microbiological testing, which may have influenced the process of consultation and the outcome of the treatment.

The risk of infectious complications after FMT depends on appropriate donor screening. This may even be more important for severely immunocompromised patients, as suggested by the cases where transfer of MDRO by FMT in neutropenic patients resulted in sepsis and death.^34^ Only 2% of potential donors were eventually eligible after extensive selection and screening. This is comparable to the donor qualification rate of 3% of a large US stool bank.^35^ Others reported higher donor acceptance rates of 10–31%.^36–38^ Unfortunately, donor exclusion criteria and screening-protocols are heterogeneous and often incomplete,^39^ underlining the need for standardization of donor screening. After initial donor acceptance, a quarter of our donors were excluded at the first quarantine screening. In addition, over half of the active donors stopped donating after six months for logistic reasons or persistent colonization by a potential pathogen. A high dropout rate was also observed in Canada; four of five approved donors were excluded during the quarantine period due to travel or acute gastro-enteritis.^40^ This demonstrates the need for a quarantine period and targeted screening on indication before faecal suspensions can be used safely. Although the identification of potential pathogens such as MDRO, norovirus or rotavirus in asymptomatic active donors is rare, the NDFB performs complete microbiological screening of the dedicated faecal suspension when the recipient is severely immunocompromised. In the future, donor selection will be even more challenging if specific donor characteristics are required for FMT treatment for indications such as ulcerative colitis or hepatic encephalopathy.^25^ In this regard, the finding that faecal suspensions with a shelf-life of 2 years at –80°C are safe and evenly efficacious for treatment of rCDI is encouraging.

In conclusion, the use of strict donor selection criteria, standardized processing and storage of FMT suspensions, and consultation by a multidisciplinary FMT-expert team, as provided by a professional stool bank, results in safe and efficacious application of FMT for rCDI. With the increasing number of reports pointing to potential beneficial effects of FMT in patients with a variety of gastro-intestinal and extra-intestinal disorders, a growing demand of FMT can be expected in the near future. Initially, experimental studies will have to be performed in a controlled setting. However, for routine clinical practice, standardised preparation, quality control and careful and long-term monitoring of outcomes and adverse events, stool banks are required. We encourage FMT centres and stool banks to utilize a multidisciplinary FMT team of experts to fill a currently existing gap, and ensure a safe and controlled application of FMT.

## References

[1] van Nood E Vrieze A Nieuwdorp M, et al Duodenal infusion of donor feces for recurrent *Clostridium difficile*. N Engl J Med 2013; 368: 407–415. 23323867 10.1056/NEJMoa1205037

[2] Hvas CL Dahl Jorgensen SM Jorgensen SP, et al Fecal microbiota transplantation is superior to fidaxomicin for treatment of recurrent *Clostridium difficile* infection. Gastroenterology 2019; 156: 1324–1332.e1323. 30610862 10.1053/j.gastro.2018.12.019

[3] Quraishi MN Widlak M Bhala N, et al Systematic review with meta-analysis: The efficacy of faecal microbiota transplantation for the treatment of recurrent and refractory *Clostridium difficile* infection. Aliment Pharmacol Ther 2017; 46: 479–493. 28707337 10.1111/apt.14201

[4] Tariq R Pardi DS Bartlett MG, et al Low cure rates in controlled trials of fecal microbiota transplantation for recurrent *Clostridium difficile* infection: A systematic review and meta-analysis. Clin Infect Dis 2019; 68: 1351–1358. 30957161 10.1093/cid/ciy721

[5] Bajaj JS Kassam Z Fagan A, et al Fecal microbiota transplant from a rational stool donor improves hepatic encephalopathy: A randomized clinical trial. Hepatology 2017; 66: 1727–1738. 28586116 10.1002/hep.29306 PMC6102730

[6] Lam WC Zhao C Ma WJ, et al The clinical and steroid-free remission of fecal microbiota transplantation to patients with ulcerative colitis: A meta-analysis. Gastroenterol Res Pract 2019; 2019: 1287493. 31178906 10.1155/2019/1287493 PMC6501134

[7] Lingen E Terveer EM van der Meulen-de Jong AE, et al Advances in stool banking. Microbiota in Health and Disease 2020; 2: e182.

[8] Kassam Z Dubois N Ramakrishna B, et al Donor screening for fecal microbiota transplantation. *N Engl J Med* Epub before print 31 October 2019. DOI: 10.1056/NEJMc1913670. 10.1056/NEJMc1913670 31665572

[9] McCune VL Quraishi MN Manzoor S, et al Results from the first English stool bank using faecal microbiota transplant as a medicinal product for the treatment of *Clostridioides difficile* infection. EClinicalMedicine 2020; 20: 100301. 32300746 10.1016/j.eclinm.2020.100301 PMC7152830

[10] Terveer EM, van Beurden YH, Goorhuis A, et al. How to: Establish and run a stool bank. *Clin Microbiol Infect* 2017; 23: 924–930. 10.1016/j.cmi.2017.05.015 28529025

[11] Kragsnaes MS Nilsson AC Kjeldsen J, et al How do I establish a stool bank for fecal microbiota transplantation within the blood- and tissue transplant service? Transfusion 2020; 60: 1135–1141. 32468608 10.1111/trf.15816

[12] Rode AA Bytzer P Pedersen OB, et al Establishing a donor stool bank for faecal microbiota transplantation: Methods and feasibility. Eur J Clin Microbiol Infect Dis 2019; 38: 1837–1847. 31273647 10.1007/s10096-019-03615-x

[13] Crobach MJ Planche T Eckert C, et al European Society of Clinical Microbiology and Infectious Diseases: Update of the diagnostic guidance document for *Clostridium difficile* infection. Clin Microbiol Infect 2016; 22: S63–S81. 27460910 10.1016/j.cmi.2016.03.010

[14] Debast SB Bauer MP Kuijper EJ. European Society of Clinical Microbiology and Infectious Diseases: Update of the treatment guidance document for *Clostridium difficile* infection. Clin Microbiol Infect 2014; 20: 1–26. 10.1111/1469-0691.12418 24118601

[15] Allegretti JR Kao D Sitko J, et al Early antibiotic use after fecal microbiota transplantation increases risk of treatment failure. Clin Infect Dis 2018; 66: 134–135. 29020157 10.1093/cid/cix684

[16] Papanicolas LE Warner M Wesselingh SL, et al Protect commensal gut bacteria to improve antimicrobial stewardship. Clin Microbiol Infect 2020; 26: 814–815. 32234452 10.1016/j.cmi.2020.03.021

[17] Wang S Xu M Wang W, et al Systematic review: Adverse events of fecal microbiota transplantation. PloS One 2016; 11: e0161174. 27529553 10.1371/journal.pone.0161174 PMC4986962

[18] Jackson M Olefson S Machan JT, et al A high rate of alternative diagnoses in patients referred for presumed *Clostridium difficile* infection. J Clin Gastroenterol 2016; 50: 742–746. 26565971 10.1097/MCG.0000000000000447 PMC4865457

[19] Shin JH Chaplin AS Hays RA, et al Outcomes of a multidisciplinary clinic in evaluating recurrent *Clostridioides difficile* infection patients for fecal microbiota transplant: A retrospective cohort analysis. J Clin Med 2019; 8: 1036. 10.3390/jcm8071036 PMC6678700 31315214

[20] Kachlikova M Sabaka P Koscalova A, et al Comorbid status and the faecal microbial transplantation failure in treatment of recurrent *Clostridioides difficile* infection – pilot prospective observational cohort study. BMC Infect Dis 2020; 20: 52. 31948404 10.1186/s12879-020-4773-x PMC6966799

[21] Ianiro G Valerio L Masucci L, et al Predictors of failure after single faecal microbiota transplantation in patients with recurrent *Clostridium difficile* infection: Results from a 3-year, single-centre cohort study. Clin Microbiol Infect 2017; 23: 337.e331–337.e333. 28057560 10.1016/j.cmi.2016.12.025

[22] Fischer M Kao D Mehta SR, et al Predictors of early failure after fecal microbiota transplantation for the therapy of *Clostridium difficile* infection: A multicenter study. Am J Gastroenterol 2016; 111: 1024–1031. 27185076 10.1038/ajg.2016.180

[23] Meighani A Hart BR Mittal C, et al Predictors of fecal transplant failure. Eur J Gastroenterol Hepatol 2016; 28: 826–830. 26934528 10.1097/MEG.0000000000000614

[24] Peri R Aguilar RC Tuffers K, et al The impact of technical and clinical factors on fecal microbiota transfer outcomes for the treatment of recurrent *Clostridioides difficile* infections in Germany. United European Gastroenterol J 2019; 7: 716–722. 10.1177/2050640619839918 PMC6545715 31210950

[25] Wilson BC Vatanen T Cutfield WS, et al The super-donor phenomenon in fecal microbiota transplantation. Front Cell Infect Microbiol 2019; 9: 2. 30719428 10.3389/fcimb.2019.00002 PMC6348388

[26] Budree S Wong WF Tu E, et al Do specific bacteria drive clinical cure in fecal microbiota transplantation for *Clostridium difficile* infection? Clinical, microbial and metabolomic characterization of universal FMT donors. Gastroenterology 2017; 152: S349.

[27] Barnes D Ng K Smits S, et al Competitively selected donor fecal microbiota transplantation: Butyrate concentration and diversity as measures of donor quality. J Pediatr Gastroenterol Nutr 2018; 67: 185–187. 29470297 10.1097/MPG.0000000000001940

[28] Tariq R Pardi DS Tosh PK, et al Fecal microbiota transplantation for recurrent *Clostridium difficile* infection reduces recurrent urinary tract infection frequency. Clin Infect Dis 2017; 65: 1745–1747. 29020210 10.1093/cid/cix618

[29] Allegretti JR Kassam Z Fischer M, et al Risk factors for gastrointestinal symptoms following successful eradication of *Clostridium difficile* by fecal microbiota transplantation (FMT). J Clin Gastroenterol 2019; 53: e405–e408. 30882536 10.1097/MCG.0000000000001194

[30] Dayananda P Wilcox MH. Irritable bowel syndrome following *Clostridium difficile* infection. Curr Opin Gastroenterol 2019; 35: 1–5. 30335629 10.1097/MOG.0000000000000490

[31] Vendrik KEW Crobach MJ Harmanus C, et al Thirteenth annual report of the National Reference Laboratory for *Clostridioides difficile* and results of the sentinel surveillance. May 2018–May 2019, https://www.rivm.nl/sites/default/files/2019-09/Annual%20report%20C.%20difficile%20reference%20laboratory%20may%202018-may%202019.pdf (2019, accessed Day Month Year).

[32] van Beurden YH de Groot PF van Nood E, et al Complications, effectiveness, and long term follow-up of fecal microbiota transfer by nasoduodenal tube for treatment of recurrent *Clostridium difficile* infection. United European Gastroenterol J 2017; 5: 868–879. 10.1177/2050640616678099 PMC5625865 29026601

[33] Perler BK Chen B Phelps E, et al Long-term efficacy and safety of fecal microbiota transplantation for treatment of recurrent Clostridioides difficile infection. *J Clin Gastroenterol* Epub before print 6 February 2020. DOI: 10.1097/mcg.0000000000001281. 10.1097/MCG.0000000000001281 32011405

[34] DeFilipp Z Bloom PP Torres Soto M, et al Drug-resistant *E. coli* bacteremia transmitted by fecal microbiota transplant. N Engl J Med 2019; 381: 2043–2050. 31665575 10.1056/NEJMoa1910437

[35] Kassam Z Dubois N Ramakrishna B, et al Donor screening for fecal microbiota transplantation. N Engl J Med 2019; 381: 2070–2072. 31665572 10.1056/NEJMc1913670

[36] Tariq R Weatherly R Kammer P, et al Donor screening experience for fecal microbiota transplantation in patients with recurrent C. difficile infection. *J Clin Gastroenterol* Epub before print 17 December 2016. DOI: 10.1097/mcg.0000000000000768. 10.1097/MCG.0000000000000768 27984397

[37] Costello SP Tucker EC La Brooy J, et al Establishing a fecal microbiota transplant service for the treatment of *Clostridium difficile* infection. Clin Infect Dis 2016; 62: 908–914. 26628567 10.1093/cid/civ994

[38] Paramsothy S Borody TJ Lin E, et al Donor recruitment for fecal microbiota transplantation. Inflamm Bowel Dis 2015; 21: 1600–1606. 26070003 10.1097/MIB.0000000000000405

[39] Lai CY Sung J Cheng F, et al Systematic review with meta-analysis: Review of donor features, procedures and outcomes in 168 clinical studies of faecal microbiota transplantation. Aliment Pharmacol Ther 2019; 49: 354–363. 30628108 10.1111/apt.15116

[40] Craven LJ Nair Parvathy S Tat-Ko J, et al Extended screening costs associated with selecting donors for fecal microbiota transplantation for treatment of metabolic syndrome-associated diseases. Open Forum Infect Dis 2017; 4: ofx243. 29255739 10.1093/ofid/ofx243 PMC5730934

